# Advances in Radiation Therapy for Unresectable Locally Advanced Non-Small Cell Lung Cancer: From Technical Advances to Immunotherapy Integration

**DOI:** 10.14789/ejmj.JMJ25-0059-R

**Published:** 2026-02-17

**Authors:** NAOYA MURAKAMI

**Affiliations:** 1 Department of Radiation Oncology, Juntendo University Graduate School of Medicine, Tokyo, Japan; 1 Department of Radiation Oncology, Juntendo University Graduate School of Medicine, Tokyo, Japan

**Keywords:** unresectable locally advanced non-small cell lung cancer, radiation therapy, immunotherapy, radiation pneumonitis

## Abstract

The management of unresectable locally advanced non-small cell lung cancer (NSCLC) has undergone significant transformation over the past three decades. This review summarizes the evolution of radiation therapy for unresectable locally advanced NSCLC, from radiation therapy alone to concurrent chemoradiotherapy, and the subsequent integration of immunotherapy or targeted therapy against driver mutations as consolidation treatment. Technical advances in radiation delivery, including the progression from two-dimensional radiotherapy to three-dimensional conformal radiotherapy (3D-CRT), followed by intensity-

modulated radiotherapy (IMRT), have improved therapeutic outcomes while reducing toxicity. The PACIFIC trial established durvalumab, an anti-programmed death ligand 1 (PD-L1) antibody, consolidation as the standard of care following concurrent chemoradiotherapy, resulting in significant improvements in survival. More recently, the LAURA trial demonstrated the efficacy of Osimertinib, an epidermal growth factor receptor tyrosine kinase inhibitor (EGFR-TKI), consolidation in EGFR-mutated NSCLC. However, radiation pneumonitis remains a potentially fatal radiation-induced toxicity that can prevent patients from receiving optimal treatment. Careful attention to dose-volume parameters, particularly lung V20Gy and mean lung dose, is essential for safe treatment delivery. Risk prediction models have been developed to identify high-risk patients, particularly those with pre-existing interstitial pneumonia. The successful transition to consolidation immunotherapy requires meticulous radiation pneumonitis management to maximize clinical benefit. This review summarizes current evidence and provides guidance for optimizing treatment approaches in this challenging patient population.

## Introduction

Unresectable locally advanced non-small cell lung cancer (NSCLC) represents approximately 30% of all lung cancer diagnoses and presents significant therapeutic challenges^1)^. Stage III NSCLC encompasses a heterogeneous group of tumors characterized by extensive local or regional spread, which often precludes surgical resection while remaining confined to the thorax. The optimal management of these patients has evolved considerably over the past three decades, driven by advances in radiation therapy techniques and systemic therapy agents.

Historically, patients with unresectable locally advanced NSCLC had limited treatment options, relying mainly on radiation therapy alone, which yielded poor outcomes with median survival times of less than 1 year^2, 3)^. The introduction of combined modality therapy, particularly concurrent chemoradiotherapy (CCRT) in the 1990s marked a significant advance, improving survival compared with radiation therapy alone and becoming the standard of care^4)^. However, despite decades of optimizations efforts, including refinements in radiation techniques and chemotherapy regimens, overall survival remained modest, with 5-year survival hovering around 15-20%, and no major breakthroughs were achieved. This long plateau persisted until the advent of immune checkpoint inhibitors. The PACIFIC trial demonstrated that consolidation therapy with durvalumab, an anti-PD-L1 antibody, after definitive CCRT significantly prolonged progression-free and overall survival, establishing a new standard of care for unresectable locally advanced NSCLC^5, 6)^.

Radiation therapy remains the cornerstone of treatment for unresectable locally advanced NSCLC. Technical advances in radiation therapy planning and delivery have enhanced the precision of treatment while reducing normal tissue toxicity. The evolution from two-dimensional radiotherapy to three-dimensional conformal radiotherapy and subsequently to intensity-modulated radiation therapy (IMRT) has enabled dose escalation to tumors while sparing critical normal structures^7, 8)^.

Despite these advances, radiation pneumonitis remains a significant concern that can limit treatment efficacy and prevent patients from receiving optimal consolidation therapy. Understanding the risk factors, predictive models, and management strategies for radiation pneumonitis is crucial for maximizing treatment outcomes^9, 10)^. This review provides an overview of the historical evolution of treatment approaches, technical advances in radiation therapy, integration of immunotherapy or targeted therapy, and strategies for managing radiation pneumonitis in patients with unresectable locally advanced NSCLC.

## Historical evolution of treatment approaches

### From radiation alone to combined modality therapy

The treatment of unresectable locally advanced NSCLC has undergone a paradigm shift from single modality approaches to integrated combined therapy. Early treatment strategies relied primarily on radiation therapy alone, which provided modest local control but limited survival benefit^2, 3)^. Subsequent recognition that microscopic disease beyond the radiation field contributed to treatment outcomes prompted the incorporation of systemic chemotherapy. In addition to addressing occult disease metastases, chemotherapy agents such as platinum compounds also act as radiosensitizers, enhancing the cytotoxic effects of radiation on tumor cells thereby improving local control. A landmark meta- analysis published in the BMJ in 1995 by the Non- small Cell Lung Cancer Collaborative Group fundamentally changed the treatment landscape^4)^. This comprehensive analysis of 52 clinical trials involving 9,387 Stage III NSCLC patients demonstrated that the addition of platinum-based chemotherapy to radiation therapy reduced cancer-related mortality by 13% (HR = 0.87, p = 0.005). This finding established chemoradiotherapy as superior to radiation therapy alone and formed the foundation for current treatment approaches.

The integration of chemotherapy with radiation therapy can be delivered in sequential or concurrent fashion with the latter combination historically considered more toxic because of increased treatment intensity due to the simultaneous administration of both treatment modalities. Sequential chemoradiotherapy involves completing chemotherapy before initiating radiation therapy, while concurrent chemoradiotherapy delivers both modalities simultaneously. The RTOG 9410 study, a pivotal randomized controlled trial involving 620 patients with unresectable stage II-III NSCLC, directly compared these approaches along with a hyperfractionated concurrent regimen^11)^. The RTOG 9410 study randomized patients to three treatment arms: sequential chemoradiotherapy with once-daily radiation (arm 1), CCRT with once-daily radiation (arm 2), and CCRT with twice-daily radiation (arm 3). The results demonstrated superior outcomes with concurrent therapy, with 5-year overall survival rates of 16% for concurrent therapy versus 10% for sequential therapy (HR = 0.81, p = 0.046). While the rate of acute grade 3-5 non-hematologic toxic effects were higher with concurrent than sequential therapy, late toxic effects were similar. This study established CCRT as the preferred approach for patients with good performance status.

The typical regimen consists of platinum-based doublet chemotherapy delivered concurrently with thoracic radiation therapy to a dose of 60 Gy in 30 fractions over 6 weeks. Common chemotherapy regimens include carboplatin and paclitaxel, cisplatin and etoposide, or carboplatin and etoposide^12)^. The choice of specific agents depends on patient factors, comorbidities, and institutional preferences.

Despite the established efficacy of concurrent chemoradiotherapy, outcomes remained suboptimal with median survival times of approximately 20-30 months and 5-year survival rates of 15-25%^1, 13)^. The high rate of distant metastases following local therapy highlighted the need for additional systemic approaches to address micro-metastatic disease.

## Technical advances in radiation therapy

### Evolution from two-dimensional to three-dimensional planning

The technical evolution of radiation therapy has played a critical role in improving outcomes for patients with unresectable locally advanced NSCLC. Early radiation therapy utilized two-dimensional planning based on plain radiographs and simple anatomical landmarks. This approach was limited by poor target definition, inadequate normal tissue visualization, and inability to optimize dose distributions.

The introduction of computed tomography (CT)- based three-dimensional conformal radiation therapy (3D-CRT) revolutionized radiation oncology practice. A large-scale analysis of the Surveillance, Epidemiology, and End Results (SEER) database examined 13,292 patients with stage III NSCLC treated with primary radiation therapy between 2003 and 2005. The study demonstrated significantly improved outcomes with 3D-CRT compared to two-dimensional planning, with 5-year survival rates of 14% versus 11%, respectively (p < 0.001)^7)^. Three-dimensional conformal radiotherapy enables precise target volume definition based on cross-sectional CT imaging and improves normal tissue sparing through conformal dose distributions. The ability to visualize target volumes and critical structures in three dimensions allows for more accurate treatment planning and delivery.

### Introduction of intensity-modulated radiation therapy (IMRT) in lung cancer radiotherapy

**Figure 1:**
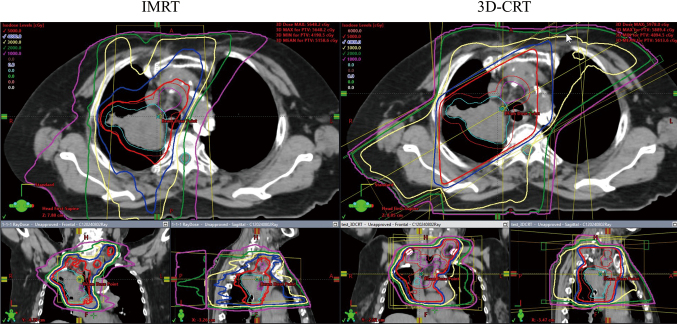
The dose distribution comparison of three-dimensional conformal radiation therapy (3D-CRT) and intensity modulated radiation therapy (IMRT) for a patient with locally advanced non-small cell lung cancer. The thick red line represents 50 Gy isodose line, and it is recognized that in IMRT, this line conforms more tightly to the target than in 3D-CRT.

Intensity-modulated radiation therapy (IMRT) represents the next evolutionary step in radiation therapy technology. IMRT utilizes computer-controlled multileaf collimators to modulate the intensity of radiation beams across the treatment fields from multiple angles, creating highly conformal dose distributions that precisely match the target volume while minimizing dose to surrounding normal tissues. The adoption of IMRT in clinical practice has increased dramatically over the past two decades, initially applied to radiotherapy for prostate cancer and head and neck cancer, and later extended to various anatomical sites including thoracic malignancies (Figure 1). An analysis of the National Cancer Database examining 4,483 patients with incompletely resected NSCLC receiving postoperative radiation therapy demonstrated a marked increase in IMRT utilization from 14.3% in 2004 to 70.7% in 2019 (P < 0.001). This increased utilization was associated with improved outcomes, with 5-year overall survival rates of 37.3% for IMRT versus 32.2% for 3D-CRT (HR 0.88, 95% CI 0.80-0.96; P = 0.003)^14)^.

The RTOG 0617 study provided important insights into both dose escalation and radiation therapy techniques. Despite CCRT, locoregional control remained poor, with locoregional progression observed in 28.1% of patients at 3 years^1)^: to address this issue, this randomized trial compared standard-dose (60 Gy) versus high-dose (74 Gy) radiation therapy in patients with unresectable stage III NSCLC^15)^. Contrary to expectations, dose escalation to 74 Gy resulted in inferior outcomes, with median survival times of 20.3 months versus 28.7 months for the 60 Gy arm (HR 1.38, 95% CI 1.09-1.76; p = 0.004). The unexpected inferior survival in the high-dose arm of RTOG 0617 has been attributed to increased radiation exposure to critical organs surrounding the tumor, particularly the heart and esophagus, leading to higher treatment-related toxicity. A secondary analysis of RTOG 0617 examined the impact of radiation therapy technique on outcomes and toxicity. The study found that IMRT was associated with a significantly reduced risk of grade ≥ 3 pneumonitis compared to 3D-CRT (odds ratio 0.41, 95% CI 0.17-0.99)^8)^. Based on this and other evidence, the 2024 American Radium Society guidelines recommend IMRT as the preferred technique for thoracic radiation therapy^16)^.

### Target volume definition and field design

**Figure 2:**
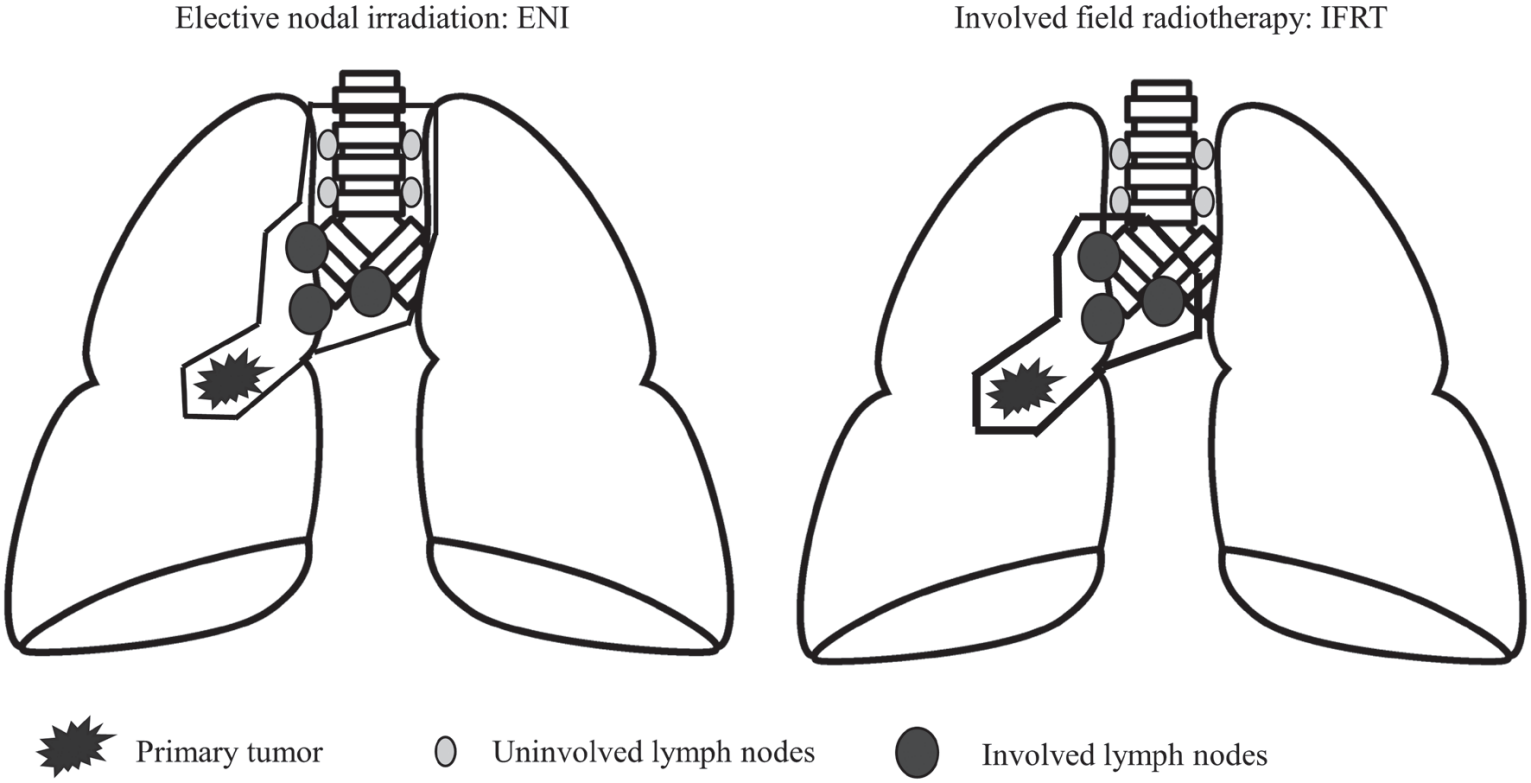
Elective nodal irradiation (ENI) encompasses the primary tumor, involved lymph nodes, and uninvolved lymph nodes that are considered high-risk for regional recurrence. Involved field radiotherapy (IFRT) includes the primary tumor and only clinically metastatic lymph nodes.

The definition of target volumes and treatment fields has evolved significantly due to advances in imaging and a better understanding of disease patterns. Two primary approaches to nodal irradiation are commonly employed: elective nodal irradiation (ENI) and involved field radiotherapy (IFRT) (Figure 2). ENI involves treating the primary tumor and elective regional nodal areas, even in the absence of radiologically evident nodal involvement, analogous to elective nodal dissection performed during surgery, whereas IFRT targets only the primary tumor and involved lymph nodes.

A comprehensive meta-analysis of 16 clinical trials involving 3,600 patients with locally advanced NSCLC compared outcomes between ENI and IFRT approaches^17)^. The analysis demonstrated superior outcomes with IFRT, with median overall survival of 24 months versus 18 months for ENI (p < 0.001). These findings support the use of IFRT approaches that minimize normal tissue irradiation while maintaining tumor control.

Modern target volume definition utilizes integrated positron emission tomography and computed tomography (PET-CT) imaging for improved tumor delineation. PET-CT provides functional information about metabolically active tumor tissue and helps distinguish tumor from atelectasis or small hilar/mediastinum lymph nodes. In a randomized, multicenter trial (PET Plan), FDG PET-guided target volume reduction using IFRT (without elective nodal irradiation) achieved non-inferior and numerically improved—locoregional control compared with conventional ENI based planning, supporting FDG PET-based IFRT as a safe and effective strategy^18)^. This enhanced imaging capability has contributed to more precise target volume definition and potentially improved treatment outcomes.

## Integration of immunotherapy in treatment paradigm

### The PACIFIC trial and durvalumab consolidation

The integration of immunotherapy into the treatment paradigm for unresectable locally advanced NSCLC represents one of the most significant advances in recent decades. The PACIFIC trial, a landmark randomized, double-blind, placebo-controlled study, established consolidation immunotherapy as the new standard of care following concurent chemoradiotherapy^6)^. The PACIFIC trial enrolled 713 patients with unresectable stage III NSCLC who had not progressed following concurrent chemoradiotherapy. Patients were randomized to receive either durvalumab, an anti-PD-L1 monoclonal antibody, or placebo for up to 12 months. The study demonstrated remarkable improvements in both progression-free survival and overall survival with durvalumab consolidation. The updated analysis of the PACIFIC trial showed a median overall survival of 47.5 months with durvalumab compared to 29.1 months with placebo (HR 0.72; 95%CI, 0.59-0.89)^5)^. The 5-year overall survival rate was 42.9% with durvalumab versus 33.1% with placebo, representing an unprecedented improvement in long-term outcomes for this patient population. Durvalumab consolidation was generally well-tolerated, with the most common adverse events being diarrhea, pneumonitis, rash, and pruritus. Immune- related adverse events occurred in approximately 24.2% of patients but were generally manageable with appropriate monitoring and intervention. The ability to deliver durvalumab consolidation depends critically on the absence of significant radiation pneumonitis following chemoradiotherapy.

### EGFR-targeted therapy: The LAURA trial

For patients with epidermal growth factor receptor (EGFR)-mutated unresectable locally advanced NSCLC, targeted therapy has emerged as an important treatment option. Approximately up to 30% of NSCLC patients harbor EGFR mutations, particularly in Asian populations. The LAURA trial investigated osimertinib, a third-generation EGFR tyrosine kinase inhibitor (EGFR-TKI), consolidation following concurrent chemoradiotherapy in patients with EGFR-mutated disease^19)^. The LAURA trial was a randomized, double-blind, placebo-controlled study that enrolled 216 patients with unresectable stage III NSCLC harboring EGFR mutations. Following concurrent chemoradiotherapy, patients were randomized to receive either osimertinib or placebo. The study demonstrated dramatic improvements in progression-free survival, with median progression-free survival of 39.1 months with osimertinib versus 5.6 months with placebo (HR 0.16; 95%CI, 0.10-0.24). Based on these compelling results, osimertinib received regulatory approval for consolidation therapy following CCRT in EGFR- mutated unresectable locally advanced NSCLC in May 2025. This represents a personalized medicine approach that tailors treatment based on tumor molecular characteristics, offering superior outcomes for appropriately selected patients.

## Management of radiation pneumonitis

### Risk factors and dose-volume parameters

**Figure 3:**
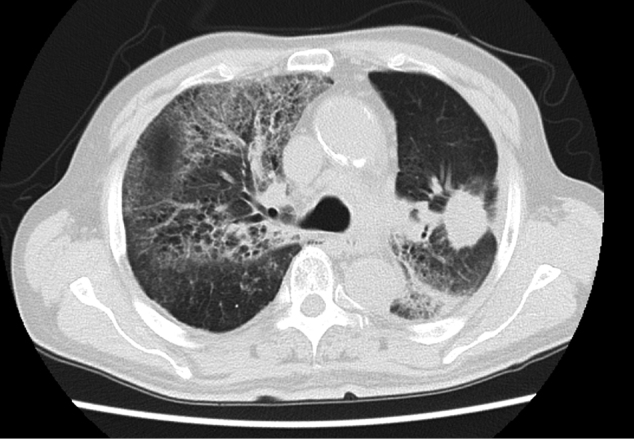
A lung CT image of a patient with locally advanced non-small cell lung cancer who had been treated with primary radiation therapy and later developed grade 4 radiation pneumonitis six months after completion of radiation therapy.

Radiation pneumonitis remains the most significant potentially life-threatening toxicity in thoracic radiation therapy and represents a critical barrier to successful treatment completion and transition to consolidation therapy (Figure 3). Understanding the risk factors and implementing appropriate prevention strategies is essential for optimizing treatment outcomes.

The lung V20Gy parameter, representing the percentage of lung volume receiving ≥ 20 Gy, has emerged as the most important predictor of radiation pneumonitis risk. A seminal study by Tsujino and colleagues established clear relationships between lung V20Gy values and the incidence of grade ≥ 2 radiation pneumonitis^9)^. The study demonstrated that patients with lung V20Gy < 20% had an 8.7% incidence of grade ≥ 2 pneumonitis, while those with V20Gy values of 21-25%, 26-30%, and > 31% had incidences of 18.3%, 51%, and 85%, respectively. Based on these findings, current guidelines recommend maintaining lung V20Gy < 30% whenever possible^20)^. Additional dose-volume parameters that correlate with pneumonitis risk include mean lung dose < 23 Gy and lung V5Gy < 60^21, 22)^. Patient- related risk factors for radiation pneumonitis include age, performance status, pre-existing pulmonary disease, smoking history, and concurrent chemotherapy. Pre-existing interstitial lung disease (ILD) represents a particularly high-risk scenario that requires special consideration and modified treatment approaches^23)^.

### Predictive risk scores for high-risk patients

Patients with pre-existing ILD represent a particularly challenging population with elevated risk of severe radiation pneumonitis. Tsujino and colleagues developed a predictive risk score (PRS) specifically for patients with interstitial lung disease undergoing concurrent chemoradiotherapy^10)^. The PRS incorporates patient age, pulmonary fibrosis score defined based on CT images focusing on percentage of honeycomb pulmonary changes, lung V20Gy, and the lung volume receiving < 5 Gy.

The PRS ranges from 0 to 17, with scores ≥ 9 identifying high-risk patients, and the model demonstrated good discrimination for predicting grade ≥ 2 radiation pneumonitis. For high-risk patients identified by the PRS, treatment modifications such as dose reduction, altered fractionation, or alternative treatment approaches should be considered. The implementation of risk prediction models in clinical practice allows for personalized treatment planning that balances efficacy and toxicity. The goal is to enable safe delivery of curative therapy while minimizing the risk of treatment-limiting toxicity.

## Future directions

### Proton beam therapy

Proton beam therapy (PBT) is emerging as a potentially safer alternative to conventional photon-based radiation therapy with its physical characteristics of confined dose distribution. In Japan, public insurance coverage for PBT was expanded in 2024 to include early-stage lung cancer (stages I-IIA), while stage III disease is not yet reimbursed under the national scheme. A large retrospective analysis in the durvalumab era from the United States showed that while there was no difference in progression-free survival or overall survival between the two arms, PBT significantly reduced unplanned hospitalization and grade ≥ 3 lymphopenia compared to IMRT, suggesting better toxicity profile due to better dose distribution^24)^. The randomized phase III RTOG 1308/NRG trial directly compares PBT versus photon CCRT for inoperable stage II-IIIB NSCLC with co primary endpoints of overall survival and cardiotoxicity/lymphopenia^25)^; patient accrual completed in 2023 and final results are pending. This head to head comparison is expected to clarify the clinical benefit of PBT for locally advanced NSCLC and may inform future reimbursement discussions.

### Personalized consolidation strategies

The future of treatment for unresectable locally advanced NSCLC is expected to increasingly embrace personalized medicine, guided by molecular biomarkers and dynamic response assessment. Beyond EGFR mutations, other targetable driver mutations such as anaplastic lymphoma kinase (ALK) rearrangements, ROS proto-oncogene 1 (ROS1) fusions, rearranged during transfection (RET) mutations, c-MET proto-oncogene (MET) exon 14 skipping mutation, v-raf murine sarcoma viral oncogene homolog B (BRAF) mutations, and other driver mutations are anticipated to become targets for consolidation therapy following CCRT^26)^. Furthermore, biomarkers such as circulating tumor DNA (ctDNA) may enable real-time response monitoring during or after treatment, facilitating adaptive strategies such as treatment de-escalation for ctDNA-negative patients or selective intensification for ctDNA-positive patients^27)^. Integration of such liquid biopsy techniques into routine clinical practice and effective targeted drugs may enable more personalized and precise treatment monitoring and adaptive treatment strategies.

## Conclusions

The evolution of the management of unresectable locally advanced NSCLC from radiation therapy alone to concurrent chemoradiotherapy and subsequently to chemoradiotherapy followed by immunotherapy consolidation has resulted in unprecedented improvements in patient outcomes.

Future progress will likely depend on continued technological innovation, biomarker development for personalized treatment selection, and the integration of novel systemic therapies. The key to success lies in maintaining the careful balance between treatment efficacy and toxicity, ensuring that patients can safely receive optimal therapy while minimizing the risk of treatment-limiting complications.

The multidisciplinary management of unresectable locally advanced NSCLC requires close coordination between radiation oncology, medical oncology, surgery, and supportive care teams. Clear communication, standardized protocols, and evidence-based treatment approaches are essential for optimizing patient outcomes in this complex clinical scenario.

## Author contributions

NM is solely responsible for the conception, data collection, and writing of this manuscript.

## Conflicts of interest statement

The author declares that there are no conflicts of interest.
